# An attention-based deep learning network for lung nodule malignancy discrimination

**DOI:** 10.3389/fnins.2022.1106937

**Published:** 2023-01-09

**Authors:** Gang Liu, Fei Liu, Jun Gu, Xu Mao, XiaoTing Xie, Jingyao Sang

**Affiliations:** ^1^Department of Interventional Radiology, Qinghai Red Cross Hospital, Xining, Qinghai, China; ^2^School of Computer Science and Technology, Department of Telecommunications, Xi’an Jiaotong University, Xi’an, China

**Keywords:** lung nodules, artificial intelligence, multimodal, malignancy, attention mechanism gate module

## Abstract

**Introduction:**

Effective classification of lung cancers plays a vital role in lung tumor diagnosis and subsequent treatments. However, classification of benign and malignant lung nodules remains inaccurate.

**Methods:**

This study proposes a novel multimodal attention-based 3D convolutional neural network (CNN) which combines computed tomography (CT) imaging features and clinical information to classify benign and malignant nodules.

**Results:**

An average diagnostic sensitivity of 96.2% for malignant nodules and an average accuracy of 81.6% for classification of benign and malignant nodules were achieved in our algorithm, exceeding results achieved from traditional ResNet network (sensitivity of 89% and accuracy of 80%) and VGG network (sensitivity of 78% and accuracy of 73.1%).

**Discussion:**

The proposed deep learning (DL) model could effectively distinguish benign and malignant nodules with higher precision.

## 1. Introduction

Lung tumors are one of the most common tumors in the world and classification of benign and malignant lung tumors is essential to the subsequent treatments. Benign lung tumors account for less than 1% of the pulmonary neoplasms (Kern et al., 2000) and a previous study indicated that wedge resection is a definitive surgery treatment for benign lung tumors (Dalouee et al., 2015). Most lung tumors are malignant and nearly 20% of cancer mortalities are caused by lung cancer (Wild et al., 2020). Since there are no apparent symptoms at early stage, people who die from lung cancer are often diagnosed at advanced stage, many effective early detection, classification, and medical management have been proposed to decrease lung cancer mortality (Naik et al., 2020).

The widespread implementations of computed tomography (CT) lung screenings have led to a massive increase in the lung nodules detected (Xing et al., 2015), however, pulmonary nodule malignancy distinction could be difficult due to human subjectivity and sometimes fatigue involved in CT image interpretations and the resulting accuracy of distinguishing malignant pulmonary nodules were only 53.1–56.3% for radiologists (Gong et al., 2019). Recently, the success of deep learning (DL) techniques in medical image analysis has prompted many investigators to employ DL in lung nodule classifications, nevertheless, the differentiation accuracies of benign, and malignant lung nodules were not satisfactory (Hua et al., 2015; Kumar et al., 2015; Guan et al., 2021). For example, Ardila et al. (2019) used a DL approach to estimate lung nodule malignancy based on changes in nodule volume with a sensitivity of merely 59.3%. Kumar et al. (2015) used convolutional neural networks (CNNs) to classify lung nodules malignancy with an accuracy of only 77.52% and Hua et al. (2015) applied the deep CNN and deep belief network (DBN) only to achieve a moderate sensitivity of 73.4% for lung nodule malignancy discrimination.

The key to the successful application of DL method is how to design a DL network architecture with strong feature extraction capabilities, since the input data are 3D CT images which not only significantly increase the computational complexity, but also many problems such as the convergence and stability of the network may occur (Qi et al., 2017; Saha et al., 2020; Dufumier et al., 2021).

In order to solve the above issues on feature extractions, attentional thinking in human vision were proposed and used in natural language processing, image classification, and other machine learning tasks. Computer vision methods based on a trainable attention mechanism could effectively and autonomously focus on the regions of interest (ROIs) for tasks, suppress irrelevant regions, and further improve the performance of DL models (Vaswani et al., 2017; Gupta et al., 2021; Zhang et al., 2021). This paper proposes a multimodal attention-based 3D deep CNN to classify lung nodule malignancy from chest CT images. The experimental results show that the proposed deep CNN model with the introduction of the attention mechanism could effectively improve the accuracy of lung nodule classification which could potentially improve image diagnosis for radiologists.

In this study, an attentional mechanism neural network architecture was designed to identify benign and malignant lung nodules in CT images, and satisfying classification performance was achieved in the experiment. The manuscript was divided into four parts: (1) the background and current situation of benign and malignant identification of pulmonary nodules based on CT imaging and the summary of this study were described. (2) The inclusion and exclusion criteria of patient cases and description of the deep neural network method proposed in this study. (3) The experimental results of benign and malignant recognition of pulmonary nodules based on the attention mechanism neural network model were analyzed. (4) The work was further discussed in detail, and the limitations of this study were given.

## 2. Materials and methods

### 2.1. Datasets

**FIGURE 1:**
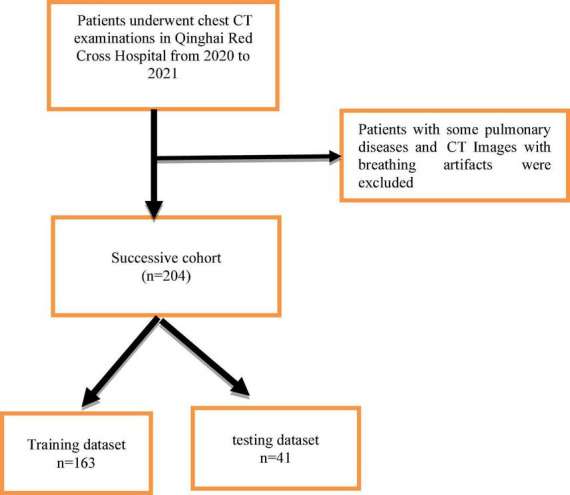
Flowchart of patients for inclusion in the successive cohort.

This single-center retrospective study included patients who visited Qinghai Red Cross Hospital for chest CT examinations from October 2020 to December 2021. Patients with chronic obstructive pulmonary disease, interstitial lesions, various types of pneumonia, and other diffuse lesions and patients with CT image breathing artifacts were excluded. All patient images were derived from the picture archiving and communication system (PACS) system and patient clinical information was obtained from the hospital medical record management system including gender, age, ethnicity, occupation, tumor history, tumor autoantibodies, tumor indicators, pathological results, and other data. A total of 204 pulmonary nodules were found in 204 cases and each patient was present with one nodule, and there were 130 benign nodules and 74 malignant nodules. Patients were randomly split into training and testing sets with a ratio of 8:2 (Figure 1). This study was approved by the Scientific Research Ethics Committee of Qinghai Red Cross Hospital, and all patients participated voluntarily and signed informed consent. Batch number (KY-2021-14).

### 2.2. Deep learning model

#### 2.2.1. 3D convolutional neural network

**FIGURE 2:**
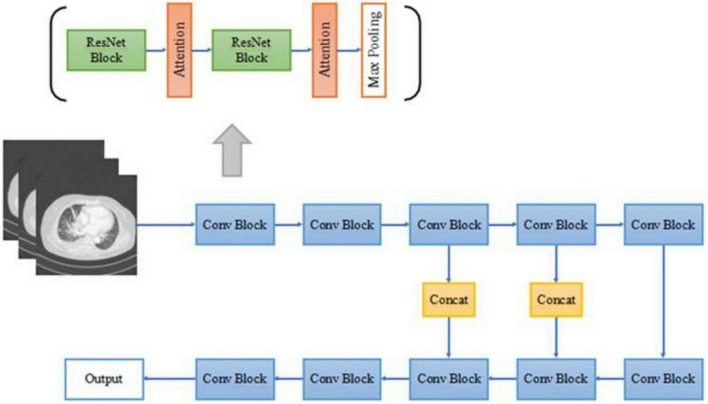
The architecture of our proposed 3D convolutional neural network (CNN).

In this paper, a deep residual network based on the attention mechanism is designed and the main network structure in this paper adopts a symmetric structure of multi-scale fusion resembling U-Net (Zhu et al., 2017; Oktay et al., 2018) consisting of residual network blocks, pooling layers, batch normalization layers, activation layers, attention mechanism gate modules, and the output layer of the region proposal network. The detailed CNN structure in this paper is shown in Figure 2.

The forward down-sampling part of the CNN consists of five 3D convolution blocks, each of which is composed of two 3D residual network convolution blocks, and a 3D maximum pooling layer following each convolution block. The pooling layer halves the scale of the image feature map, realizes the down-sampling operation of the image through pooling, extracts features, and reduces parameters for subsequent convolution operations. The deconvolution lifting part of the CNN consists of three convolution blocks and a region proposal network output layer. After the feature map is extracted from the convolution block, the deconvolution operation is used to improve the scale of the image, forming a similar structure of U-Net. Deconvolution is a convolution operation used to increase the size of the feature map, which is a kind of trainable up-sampling. After each convolution block, the image feature map scale is multiplied by 2. The splicing part in the middle is used to fuse the context information of the image, combining the low-level abstracted features with the high-level abstracted features to generate more features effectively, and is also a very important part of the approximate U-Net structure. All convolutional blocks in the network sample the same 3D residual convolution block as above, as in the forward down-sampling part.

**FIGURE 3:**
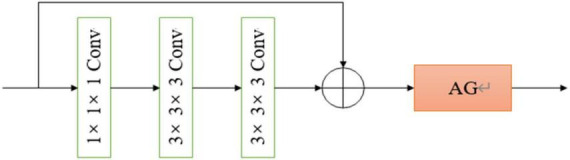
The architecture of our proposed 3D convolutional neural network (CNN).

All convolutional blocks in the network are composed of 3D versions of residual network convolution blocks, including two 1 × 1 × 1 convolution kernels, and a 3 × 3 × 3 convolution kernels, after each convolution kernel is the ReLU activation function and batch normalization, compared with the two 3 × 3 × 3 convolution kernels, the number of parameters is almost reduced by half, while the performance of the two networks is almost the same. The 3D residual network convolution block structure used in this paper is shown in Figure 3, where AG represents the attention mechanism module used.

#### 2.2.2. Attention mechanism gate module

**FIGURE 4:**
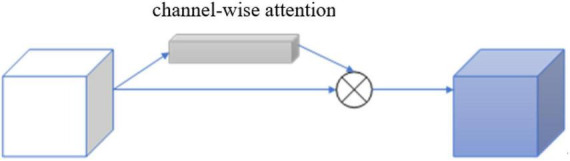
Structure of 3D residual block with attention gate.

Standard CNN models usually result in feature maps from repeated convolutions, down-sampling, and non-linear activations. The attention mechanism model can assign significant weights to task-related feature maps within the acceptable computational overhead based on existing deep CNN models. In order to improve the quality of feature maps generated by CNNs, this paper, a trainable 3D attention mechanism gate module and it was integrated into the CNN above. The 3D attention mechanism gate structure is shown in Figure 4.

The attention factor ranges from 0 to 1 and is used to identify relevant ROIs for existing image tasks and to prune and suppress irrelevant features, retaining only task- related activations and resampling the feature map. The intermediate feature map F ∈ *RL* × *W* × *H* × *C* output was obtained by a convolution operation of arbitrary size, where *RL* is the length of the 3D image, *W* is the width, *H* is the height, and *C* is the number of 3D image channels.

Each channel of the image feature map can be regarded as a feature generator (Zeiler and Fergus, 2013), and the channel-based attention gate could focus on the parts of the image channel that are meaningful to the task. To efficiently compute the attention factor required to generate channels, squeeze and excitation networks (SENets) initiated by Jie et al. (2017) was proposed to map the spatial dimension of the input feature, and additional adaptive mean pooling is added in the case of adaptive mean pooling. Max pooling was applied to enhance the expressiveness of feature maps since standard pooling technology could only obtain the desired pooling result by adjusting the pooling step size, while adaptive pooling is a pooling technology with a fixed size output.

**FIGURE 5:**
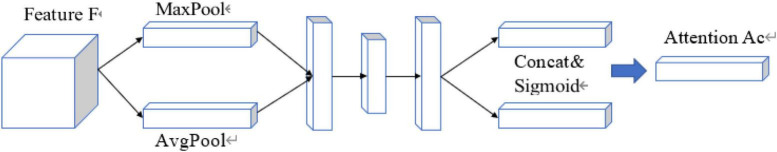
Structure of 3D attention mechanism gate module.

After obtaining the result of channel “squeeze,” the multi-hidden layer neural network was “stimulated” using an autoencoder structure with shared parameters, and then two-part pooling results were combined and finally the attention factor is obtained using the sigmoid activation function. The calculation formula of the channel attention mechanism gate is shown:


(1) A⁢c=σ⁢(M⁢L⁢P⁢(A⁢V⁢g⁢P⁢o⁢o⁢l⁢(F))+M⁢L⁢P⁢(M⁢a⁢x⁢p⁢o⁢o⁢l⁢(F)))

and its detailed structure is shown in Figure 5.

### 2.3. Experimental parameter settings

We employ PyTorch to implement our method, the version is 3.8.3 and the training and inference processes were performed on 4 NVIDIA TITAN V.

## 3. Results

### 3.1. Evaluation metrics

In terms of model evaluation metrics, we mainly deployed the accuracy and sensitivity for pulmonary nodule discriminations. The calculation of the accuracy metric was shown:


(2) $$\text{Accuracy}=\frac{\text{TP}+\text{TN}}{\text{TP}+\text{TN}+\text{FP}+\text{FN}}$$

Which was mainly used to evaluate the model’s capability of malignant pulmonary nodule judgment in overall nodules.

The calculation of sensitivity was shown:


(3) $$\text{Sensitivity}=\frac{\text{TP}}{\text{TP}+\text{FN}}$$

The sensitivity metric mainly reflects the model’s ability to correctly identify malignant pulmonary nodules in the actual malignant nodules (TP: true positive, TN: true negative, FP: false positive, FN: false negative).

### 3.2. Analysis of results

A total of 204 cases were randomly divided into training data of 163 cases (80%) and testing data of 41 cases (20%) and 10-fold cross-validation method is used to verify the classification sensitivity and accuracy of our proposed model.

The averaged results of 10-fold cross-validation of our proposed model are shown in Table 1 and compared with traditional ResNet and VGG network using the same 10-fold cross-validation based on the same dataset. As shown in the table, the newly proposed model after adding the 3D attention mechanism gate achieved better sensitivity and accuracy in distinguishing malignant nodules than ResNet and VGG network.

**TABLE 1:** Comparison of the classification results of malignant lung nodules with different methods.

Methods	Accuracy (%)	Sensitivity (%)
ResNet	80	89
VGG	73.1	78
Ours	81.6	96.2

**FIGURE 6:**
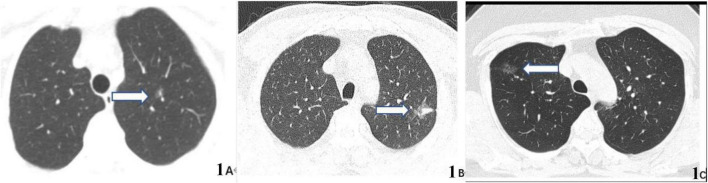
CT images of three typical lung nodule types correctly distinguished by our proposed model while wrongly determined by radiologist. **(A)** 59-year-old woman, CT scan of the chest showing a ground glass nodule in the upper lobe of the left lung with post-operative pathological result of adenocarcinoma *in situ* (AIS) (arrow). **(B)** 63-year-old man, CT scan of the chest showing a mixed ground glass nodule in the upper lobe of the left lung with post-operative pathological result of invasive adenocarcinoma (IA) (arrow). **(C)** 36-year-old man, CT scan of the chest showing a pure ground-glass nodule in the upper lobe of the right lung, which suggests an inflammatory lesion (arrows) owing to its disappearance on reexamination 3 months later.

Figure 6 shows three typical pulmonary nodules cases including adenocarcinoma *in situ* (AIS), invasive adenocarcinoma (IA), and inflammatory lesion were accurately distinguished by our model (case A and case B nodules were malignant, case C was benign) while falsely interpreted by radiologist.

## 4. Discussion

This paper proposes a lung nodule classification method based on the attention mechanism gate which combines spatial and channel attention with two different granularities and levels of feature enhancement, and the effectiveness of this method was validated. The 10-fold cross-validation results show that the average accuracy of the proposed method applying 3D attention mechanism could reach 81.6%, surpassing the traditional ResNet method of 80% and VGG network of 73.1%. The averaged sensitivity of our model in distinguishing malignant nodules from benign nodules is 96.2%, which is much higher than that derived from ResNet (89%) and VGG (78%) network.

The three typical cases presented above could not be accurately distinguished by radiologist since the first two cases (Figures 6A, B) were ground glass nodules without apparent malignant features and the last case (Figure 6C) was pure ground glass nodule without obvious benign features. So it is speculated that the attention mechanism DL model could clasp relevant imaging feature information while ignore non-critical imaging feature information more effectively to further improve the discrimination sensitivity and accuracy.

The first limitation of our study is that external public data such as LUNG16 were not used for testing, which cannot fully reflect the effectiveness of our method. Therefore, in future work, we will connect with external data to further verify the reliability of this method. Meanwhile, we will try to use a classifier based on fuzzy logic to identify benign and malignant pulmonary nodules (Davoodi and Hassan Moradi, 2018; Kumar et al., 2018). Another limitation is that classification results for different lung nodule subtypes (such as ground-glass nodules and non-ground glass nodules) were not explored and will be conducted in future work. We think that future research should also focus on developing and validating simpler nodule evaluation algorithms by incorporating emerging diagnostic modalities like molecular signatures, biomarkers, and liquid biopsies (Gaga et al., 2019), which would provide great aid to both researchers and medical practitioners.

## Data availability statement

The original contributions presented in this study are included in the article/supplementary material, further inquiries can be directed to the corresponding author.

## Ethics statement

The studies involving human participants were reviewed and approved by the Research Ethics Committee of Qinghai Red Cross Hospital. The patients/participants provided their written informed consent to participate in this study.

## Author contributions

GL and FL contributed to the conception and design of the study. XX, JS, and XM organized the database. XM and FL performed the statistical analysis. FL and JG wrote the first draft of the manuscript. XM, XX, and JS wrote sections of the manuscript. All authors contributed to the manuscript revision, read, and approved the submitted version.
