# Corrigendum: An attention-based deep learning network for lung nodule malignancy discrimination

**DOI:** 10.3389/fnins.2023.1357511

**Published:** 2024-01-12

**Authors:** Gang Liu, Fei Liu, Jun Gu, Xu Mao, XiaoTing Xie, Jingyao Sang

**Affiliations:** ^1^Department of Interventional Radiology, Qinghai Red Cross Hospital, Xining, Qinghai, China; ^2^School of Computer Science and Technology, Department of Telecommunications, Xi'an Jiaotong University, Xi'an, China

**Keywords:** lung nodules, artificial intelligence, multimodal, malignancy, attention mechanism gate module

In the published article, there was an error in the Funding statement. The Funding statement is incomplete. The correct Funding statement appears below.

